# On the Sterilisation of the Hands

**Published:** 1904-12

**Authors:** 


**On the Sterilisation of the Hands.** By C. Leedham-Green, M.B., F.R.C.S. Pp. 102. London: Simpkin, Marshall, Hamilton, Kent & Co. Ltd. 1904.

There is no part of the technique of surgeons more varied than the methods by which they attempt to sterilise their hands. A vast amount of good work has been done on this subject in many countries, and still there is no agreement. The reason of this is not hard to see if one reads this monograph. No method is satisfactory. The writer has approached the subject with great and honest care and with an unbiased mind. He has multiplied his experiments, and has used numerous different hands for trial. He concludes that soap and water alone is useless, in fact worse than useless. Even if sand be added the results are no better, however long the washing is continued. Treatment afterwards with turpentine, aqueous solutions of carbolic, lysol, perchloride and binodide of mercury give no better results. Permanganate of potash and oxalic acid are quite inadequate. Ether is not mentioned. Alcohol possesses a remarkable power of sterilising the hands far surpassing that of all other agents, but it must be used for at least five minutes, and a rough hand can in no way be cleaned. The most efficient method of all is to use soap and water and sea sand for five minutes, methylated spirit for three minutes, and 20 per cent. sublimate alcohol (1 in 1,000), rub dry. But even this is not reliable, so gloves should be worn when possible.

